# Take it personal! Development and modelling study of an indicated prevention programme for substance use in adolescents and young adults with mild intellectual disabilities and borderline intellectual functioning

**DOI:** 10.1111/jar.12808

**Published:** 2020-09-29

**Authors:** Esmée P. Schijven, Joanneke E. L. VanDerNagel, Roy Otten, Jeroen Lammers, Evelien A. P. Poelen

**Affiliations:** ^1^ Research and Development Pluryn Nijmegen The Netherlands; ^2^ Behavioral Science Institute Radboud University Nijmegen Nijmegen The Netherlands; ^3^ Centre for Addiction and Intellectual Disability (CAID) Tactus Deventer The Netherlands; ^4^ Nijmegen Institute for Scientist‐Practitioners in Addiction Radboud University Nijmegen Nijmegen The Netherlands; ^5^ Aveleijn Borne The Netherlands; ^6^ Human Media Interaction Faculty of Electrical Engineering, Mathematics, & Computer Science University of Twente Enschede The Netherlands; ^7^ REACH Institute Arizona State University Tempe AZ USA; ^8^ Trimbos Institute Netherlands Institute of Mental Health and Addiction Utrecht The Netherlands

**Keywords:** alcohol use, borderline intellectual functioning, drug use, intervention, Intervention Mapping protocol, mild intellectual disabilities

## Abstract

**Background:**

This paper describes the theory and development of Take it personal! an indicated prevention programme aimed at reducing substance use in individuals with mild intellectual disabilities and borderline intellectual functioning.

**Method:**

The process of the development of Take it personal! followed the steps of the Intervention Mapping protocol. Take it personal! is based on the theory that personality traits are an important construct to understand substance use (14–30 years old). A small modelling study was conducted with six adolescents to examine the feasibility, user‐friendliness and potential effectiveness of the intervention.

**Results:**

The results showed that the intervention has good feasibility and user‐friendliness. Post‐intervention evaluation of frequency, binge drinking and problematic use indicated that use was lower than at pre‐intervention.

**Conclusions:**

Take it Personal! can be a promising preventive intervention designed to reduce substance use in individuals in this target group. A larger scale study is needed to draw further conclusions.

## 1 INTRODUCTION

Substance use among individuals with a mild intellectual disability or borderline intellectual functioning (MID‐BIF; IQ between 50 and 85 and limitations in social adaptive skills; American Psychiatric Association, 2013) is a pressing problem. In treatment facilities in the Netherlands, individuals with mild intellectual disabilities and individuals with borderline intellectual functioning are commonly considered to belong to the same target group (de Wit, Moonen, & Douma, 2011) as both groups suffer from the same problems as a consequence of impaired intellectual and adaptive functioning (Baglio et al., 2014; Hassiotis et al., 2008; Peltopuro, Ahonen, Kaartinen, Seppälä, & Närhi, 2014). Since people with MID‐BIF lead more ordinary and less restricted lives now than they did a decade ago, they became more at‐risk to be exposed to social and environmental pressures encouraging them to adopt behaviours that negatively affect their health, such as exposure to stressful events and alcohol and drugs (Kepper, VanDen Eijnden, Monshouwer, & Vollebergh, 2014; Taggart, McLaughlin, Quinn, & Milligan, 2006). Eventually, this level of exposure may lead to substance use disorders and related problems (Burgard, Donohue, Azrin, & Teichner, 2000; Kepper et al., 2014; Taggart et al., 2006).

Studies have shown that substance use is common in adolescents and young adults with MID‐BIF, especially among those with behavioural problems and co‐morbid psychopathology who are admitted to treatment facilities (VanDerNagel et al., 2017; Van Duijvenbode et al., 2015). Prevalence rates show that 75%–85% of adolescents with MID‐BIF and severe behavioural problems who are admitted to treatment facilities show lifetime alcohol use or use alcohol on a regular basis, and 25%–50% of these adolescents use drugs (in particular, cannabis) on an occasional or regular basis (daily or weekly use). For most cannabis users, cannabis is a part of their daily routine (Bransen, Schipper, & Blekman, 2009). Adolescents with a MID‐BIF are at a higher risk for substance use disorders compared to their non‐disabled peers (Burgard et al., 2000; McGillicuddy, 2006; Van Duijvenbode et al., 2015).

The consequences of substance use are more negative for adolescents with MID‐BIF than for their non‐disabled peers, as alcohol and drugs disproportionally affect the physical and mental health of people with MID‐BIF, leading to more severe behavioural and social difficulties (Barrot & Paschos, 2006; Didden, 2017; To, Neirwynck, Vanderplasschen, VanHeule, & VanDerVelde, 2014). Substance use by adolescents with MID‐BIF causes various problems, including social, mental, behavioural, criminal and financial (Taggart et al., 2006). Moreover, adolescents with MID‐BIF are at an increased risk for substance use disorders (Burgard et al., 2000; Taggart et al., 2006).

Because of these issues, early intervention *before* the onset of substance use disorders is of vital importance. Therefore, it is necessary to develop preventive interventions and adapt the existing ones specifically to the needs and capabilities of adolescents with MID‐BIF (Kiewik, VanDerNagel, Engels, & de Jong, 2017). Practitioners from treatment facilities for people with MID‐BIF are often inexperienced with the treatment of substance use. In the past decade, attention to substance use in intellectual disability care has increased, leading to the development of several interventions (Kiewik et al., 2017). However, the need for interventions tailored specifically to this group is still high, as effective prevention programmes for adolescents with MID‐BIF are rare (Lawrence, Kerr, Darbyshire, Middleton, & Fitzsimmons, 2009).

Current prevention programmes for adolescents with MID‐BIF focus mainly on increasing knowledge of alcohol and drugs and the negative consequences of substance use (Kerr, Lawrence, Darbyshire, Middleton, & Fitzsimmons, 2013; Kiewik, VanDerNagel, Kemna, Engels, & de Jong, 2015). Increasing knowledge of substance use prevention may be ineffective for adolescents with MID‐BIF, because that the concept of behavioural change in people with MID‐BIF is not likely to be a well‐considered and rational process.

Studies have shown that adolescents with MID‐BIF already have enough knowledge of substances and their negative consequences (Kiewik et al., 2015; VanDuijvenbode et al., 2015). An intervention should, therefore, focus on adolescents and young adults who already use alcohol and other substances and teach them useful competences to prevent further development of a substance use disorder (i.e. indicated prevention).

The present paper describes the theory and development of Take it personal! an indicated prevention programme for reducing substance use in adolescents with MID‐BIF and severe behavioural problems. Take it personal! is based on the theory that personality traits are important in understanding substance use. Studies have distinguished four personality profiles based on impulsivity, sensation seeking, anxiety sensitivity and negative thinking (assessed with the Substance Use Risk Profile Scale, SURPS; Poelen, Schijven, Otten, & Didden, 2017; Woicik, Stewart, Phil, & Conrod, 2009), all of which are known to be associated with substance use (disorders) in the general population (Conrod, Comea, & Maclean, 2006; Woicik et al., 2009) and among individuals with MID‐BIF (Poelen et al., 2017). In addition, this paper contains an assessment of its feasibility, user‐friendliness and potential effectiveness based on the results of a small modelling study.

## 2 METHODS

### 2.1 Intervention mapping

Take it personal! is an indicated prevention programme aimed primarily at reducing substance use (alcohol, cannabis and illicit drug (other drugs that are more addictive, more potent and more toxic, such as cocaine in any form, e.g. crack; club drugs, e.g. ecstasy; hallucinogens, e.g. LSD or opioids, e.g. heroin) and preventing the onset of substance use disorder. The secondary aims of Take it personal! were to decrease the intention to use alcohol, cannabis and/or illicit drugs in the future and to decrease internalizing and externalizing behavioural problems. Intervention Mapping (IM) was used to guide the development of this intervention.

IM is a useful tool for theory and evidence‐based development of health promotion interventions (Bartholomew, Parcel, Kok, & Gottlieb, 2001), and it has been shown to be useful in developing substance use interventions (e.g. Dupont, Lemmens, Adriana, VanDeMheen, & DeVries, 2015) and interventions for people with MID‐BIF (Schaafsma, Stoffelen, Kok, & Curfs, 2013). The IM process starts with a needs assessment followed by 5 steps: (i) definition of programme objectives, (ii) selection of intervention methods and strategies, (iii) development of programme plan and pilot study of the intervention, (iv) adaptation and implementation plan and (v) evaluation plan.

The intervention aims to trigger a behavioural change in adolescents and young adults (age 14–30 years) with MID‐BIF and severe behavioural problems (Schijven, VanDerNagel, Lammers, & Poelen, 2014). Take it personal! targets adolescents and young adults who (i) use alcohol, cannabis or illicit drug use at the stage of experimental use to a mild substance use disorder according to the DSM‐5 (American Psychiatric Association, 2013), and (ii) who can be placed in one of the four personality high‐risk groups (SS, IMP, AS or NT). A moderate to severe substance use disorder according to the DSM‐5 was a contraindication. Co‐morbid behavioural problems were no contraindication but for individuals experiencing extreme aggression problems or suffering from psychotic symptoms, the intervention is not appropriate.

Take it personal! is based on the theory that personality traits are important in understanding substance use. Studies have distinguished four personality profiles based on impulsivity, sensation seeking, anxiety sensitivity and negative thinking, all of which are known to be associated with substance use (disorders) in the general population (Conrod et al., 2006; Woicik et al., 2009) and among individuals with MID‐BIF (Poelen et al., 2017). Each personality profile is also associated with specific substance misuse patterns and vulnerability to specific forms of co‐morbid psychopathology in adolescents (Conrod & Woicik, 2002). In individuals with MID‐BIF, substance use is often related to these forms of co‐morbid psychopathology and these associated profiles (Poelen et al., 2017; VanDuijvenbode et al., 2015) and therefore interventions targeting these four personality profiles are highly relevant. Impulsive adolescents lack the ability to delay a behavioural response when faced with immediate reinforcement (Conrod & Woicik, 2002). Sensation seeking is associated with increased risk‐taking or reckless behaviours among teens, such as shoplifting, unprotected sex, dangerous driving, and alcohol and other drug use (Arnett, 1994). Sensation seekers tend to be heavy drinkers at an increased risk for adverse drinking consequences (Conrod et al., 2006). Highly anxious sensitive persons show increased levels of drinking (Stewart, Loughlin, & Rhyno, 2001), are more responsive to the anxiety‐reducing effect of alcohol, are more likely to use alcohol to cope with negative feelings (Comeau, Stewart, & Loba, 2001) and are at risk for problem drinking (Conrod, Pihl, & Vassileva, 1998). They often cope with their negative feelings by using a combination of withdrawal (from social situations), dependence (on others to make them feel better), or alcohol and/or drug use. People with high levels of negative thinking or depression often have specific motives for substance use (Blackwell, Conrod, & Hansen, 2002) that help them cope with negative feelings (Malmberg et al., 2010). The intervention Take it personal! aims at giving adolescents and young adults with MID‐BIF confidence to deal with their personality traits and associated motives for excessive substance use. For each personality profile, a different intervention was developed and participants join the intervention with participants with a similar personality profile. The design and construction process of the intervention is identical for each profile, but accents are different and focus on the particular personality profiles. For instance, participants with the personality profile impulsive behaviour learn to think before they act, and practices for participants with an anxiety sensitivity profile focus on relaxation exercises and overcoming fears.

The intervention is based on methods and practical strategies of motivational interviewing (MI) and cognitive behavioural therapy (CBT), which have been proven to be effective in decreasing alcohol and drug use among non‐disabled adolescents (Conrod, Castellanos‐Ryan, & Mackie, 2011; Lammers et al., 2015; Riper et al., 2014). MI is a collaborative person‐centred technique to elicit and strengthen motivation for change (Miller, 1983). It focuses on exploring and resolving ambivalence through motivational processes within the individual that facilitate change. MI is considered an evidence‐based practice in the treatment of individuals with substance use disorders (Riper et al., 2014), and it has been adapted for persons with MID‐BIF (Frielink & Embregts, 2013). CBT is an action‐oriented form of psychosocial therapy, which assumes that maladaptive or ineffective thinking patterns cause maladaptive behaviour and negative emotions (maladaptive behaviour is behaviour that is counter‐productive or interferes with everyday living). CBT has become increasingly available to people with intellectual disabilities, and studies have shown positive effects of CBT on the reduction of mental health problems, anger and aggression in people with MID‐BIF (Didden, Korzilius, Oorsouw, & Sturmey, 2006; Vereenooghe & Langdon, 2013). Take it personal! is based on simplified CBT methods and practical strategies and does not comprise a complete CBT protocol. Using CBT with individuals with MID‐BIF requires adaptations such as using simplified language, working in small steps and carefully applying the methods to home and school/work situations, shorter sessions and additional explanation. In addition, CBT in Take it personal! is simplified and does not teach participants to distinguish feelings and thoughts that precede SU (Didden et al., 2006).

Another technique especially added for the target group in this intervention is psychomotor therapy. Psychomotor therapy is a commonly used method for adolescents with MID‐BIF, and practitioners have good experiences with daily body movement exercises conducted through sports and physical education. The psychomotor therapist focuses on problems that emerge in movement behaviour, body language, physical tension, posture, body sensations and body experience. First, results of effectiveness of psychomotor therapy among adolescents with MID‐BIF show positive findings (Bellemans, 2019). The proposed intervention will incorporate psychomotor therapy exercises into each group session to complement the CBT.

Take it personal! comprises three main components: (i) psycho‐education, (ii) behavioural coping skills and (iii) cognitive coping skills. At the start, the intervention focuses on psycho‐education addressing the participants' personality profile and related problematic coping behaviour, like substance use or aggression. Psycho‐education was adapted to the level of intellectual functioning by using games, visual support and the use of daily life experiences. The confident (the role of the confident is explained in the following paragraph) has a supporting role by bringing up these daily life experiences. In this phase, the participants will become familiar with their personality profile and learn to deal with their personality through exercises. Daily life experiences and subsequent physical, cognitive and behavioural reactions will be analysed. Moreover, they will set individual goals, which they will encounter during the training. The participants will identify personality‐specific thoughts that lead to problematic behaviour. For example, the intervention aimed at adolescents with the personality profile “Impulsive” will focus on thinking before taking action. Simultaneously, the participants will be trained to use cognitive restructuring techniques to counter such thoughts. They will be asked to edit a personalized “changing plan” to deal differently with their problematic and risky behaviour. Table 1 shows a schematic overview of the themes and objectives per week.

**Table 1:** Schematic overview of the themes and objectives of Take it personal! per week

Theme's	Objectives
Week 1 About you	Meeting each other, establishing security and trust. Room and time to make participants feel at ease. Explaining the personality profile and recognition of participants' own situation in a positive way. A small step to the consequences for behaviour.
Week 2 Effect	Setting individual long‐term goals for the intervention and show the participants that small steps are needed to achieve these goals. Understanding that risky behaviour can stand in the way of achieving these long‐term goals.
Week 3 What precedes?	Identification of thoughts and feelings that precede behaviour and learning to recognize these signals. Learning participants to realize that there is a moment to make a different choice.
Week 4 The challenge	Participants make a personalized change plan that helps them to cope differently with their behaviour. This includes specific actions for the participant and people in his/her environment to support him/her.
Week 5 Evaluating the change plan	Evaluating the change plan and if necessary adapt it. Specific focus on success and positive reinforcement.

The intervention will involve five group sessions and six individual sessions spread across 6 weeks. Individual sessions will last 30 min, and group sessions will last 45 min (depending on intellectual functioning, there was the possibility to spend more or less time per group or individual session). During the individual sessions, participants may invite a confidant for support. The confidant is someone from the professional network of the participant (e.g. a caregiver), who has an explicit role in establishing the transfer of training to everyday life situations. The design of the intervention with individual sessions and group sessions was specifically modified for this target group. Personality‐targeted interventions for adolescents without MID‐BIF only comprise group sessions (i.e. Conrod et al., 2011; Lammers et al., 2015). In each session, trainer, participant and confidant will go through the group sessions of that week. With this approach, the participants are better prepared for the group session. Besides, it provides repetition, which is necessary for effective interventions for individuals with MID‐BIF (de Wit et al., 2011). Participants use a workbook during the sessions for different exercises, such as the “changing plan.” The workbook contains minimal text and is especially designed with individuals from the target group together with graphic designers.

### 2.2 Modelling study

#### 2.2.1 Design

Based on the SURPS (Poelen et al., 2017; Woicik et al., 2009), participants were enrolled in two intervention groups (*n* = 4 impulsive behaviour and *n* = 2 sensation seeking). Participants received either the impulsive behaviour intervention or the sensation seeking intervention, and all participants were associated with a treatment centre for their behavioural problems in the Netherlands. A pre‐test (2 weeks before the intervention) and a post‐test were conducted (2 weeks after the last session) to measure potential intervention effects. To measure feasibility and user‐friendliness, participants and trainers were asked to fill in questionnaires 2 weeks after the last session.

#### 2.2.2 Participants and procedure

Seven adolescents with MID‐BIF with severe behavioural problems placed in residential care were invited to participate. Based on the SURPS (Poelen et al., 2017; Woicik et al., 2009), four adolescents were enrolled in the impulsivity intervention and three adolescents in sensation seeking. One of the participants ran away from the treatment centre and dropped out, leading to an ultimate sample size for the pilot study of six participants. Other than this participant, there was no further dropout of any sessions. The participants were three boys and three girls between 15 and 19 years of age (*M* = 16.7; *SD* = 1.5). The average level of intelligence (according to the personal files) of the participants was 70.2 (*SD* = 6.4) with an IQ ranging from 58 to 77. Three participants were diagnosed with ADHD, with one having a co‐morbid conduct disorder. Two participants were diagnosed with attachment disorder and one with symptoms of depression. Therapists were a psychologist and psychomotor therapist who had received specific training on Take it personal! prior to the start of the study, including training in CBT, MI and the theoretical background of the intervention. A pre‐test was conducted 2 weeks before the start of the intervention and a post‐test 2 weeks after the last session.

#### 2.2.3 Measurements

##### Feasibility

Feasibility in this study was investigated as it is pivotal to know how trainers experienced providing the intervention. This is helpful for implementation and adherence of trainers and participants to the protocol (Carney, Johnson, Carrico, & Myers, 2020). To assess feasibility, trainers were asked to complete an open‐ended questionnaire after the intervention. The first section of the questionnaire consisted of general questions, for example, “Do you think the training connects with the target group? Why do you think so?” or “Is there sufficient variation between talking and doing things during the sessions?” In the second part, the trainers were asked to answer the following questions for each assignment in the training, “Did you achieve the purpose of the assignment during the sessions?” “Were the assignments matching with the target group?” and “Why do you think so?”

##### User‐friendliness

User‐friendliness was assessed to obtain feedback about the intervention in this study, because it is important as user‐friendliness prevents high numbers of dropout (a lack of user‐friendliness evokes higher levels of stress; Pauly et al., 2018). Questions were about how much they liked the intervention, whether they thought they had learned anything from the intervention, whether they thought the counsellor had added value, how they liked the design of the intervention, and whether they thought there was a good balance in talking en doing things during the intervention. To measure user‐friendliness, the participants were asked to fill in an evaluation questionnaire after the intervention. The first part of the questionnaire consisted of general questions about the intervention; examples are, “I liked the intervention” or “I liked the design of the workbook.” Participants could answer these questions on a 4‐point scale illustrated with figures of thumbs up or down. Answer categories ranged from (1) “not right at all” (two thumbs down) to (4) “totally right” (two thumbs up). In the second part, the participants were asked about their opinion regarding specific assignments, for example, “I liked the Bingo game.” They could answer those questions using the same 4‐point scale.

##### Potential effectiveness

To assess alcohol and drug use, we administered the Substance Use and Misuse in Intellectual Disability Questionnaire (SumID‐Q; VanderNagel, Kiewik, Van Dijk, De Jong, & Didden, 2011; VanDuijvenbode, Didden, Korzilius, & Engels, 2013) at the pre‐test and at the post‐test 2 weeks after the last session. The SumID‐Q was especially developed to determine the substance use among individuals with MID‐BIF. Similar items were included to measure alcohol, cannabis and illicit drug use. Primary outcomes for substance use were frequency of use, binge drinking and severity of substance use. The frequency of use was assessed with the item, “How often do you use alcohol?” Response options ranged from (1) “less than once a month” to (4) “almost every day” (VanDerNagel et al., 2011; VanDuijvenbode et al., 2013). Binge drinking was assessed with the question, “How often do you drink more than 6 glasses?” The answer categories ranged from (1) “never” to (5) “almost every day” (VanDerNagel et al., 2011; VanDuijvenbode et al., 2013). The severity of alcohol use was assessed using the Alcohol Use Disorders Identification Test (AUDIT; Babor, Higgins‐Biddle, Saunders, & Monteiro, 2001), which was incorporated in the SumID‐Q. The severity of drug use was assessed using the Drugs Use Disorders Identification Test (DUDIT; Berman, Bergman, Palmstierna, & Schlyter, 2003). The items measured the frequency and quantity of use, dependency and problems related to use. An example of an item is, “How often did you regret drinking/drug use?” Response options ranged from (1) “never” to (5) “almost every day.” Each scale consisted of 10 items (VanDerNagel et al., 2011). Total scale scores were calculated by summing the items. These outcomes measures have shown psychometric qualities with respect to validity and reliability (Poelen et al., 2017; VanDuijvenbode et al., 2013).

#### 2.2.4 Analysis

To provide information on the feasibility and user‐friendliness of Take it personal! descriptive analyses were conducted based on data derived from a combination of Likert‐scale questions and open‐ended questions. To test the potential effectiveness, we operationalized goals for alcohol, cannabis and illicit drug use (Schijven, Engels, Kleinjan, & Poelen, 2015). We examined whether there was a decrease in use of alcohol, cannabis and illicit drug use in absolute numbers in the last month use, weekly use and the AUDIT (Babor et al., 2001) and DUDIT (Berman et al., 2003).

## 3 RESULTS

### 3.1 Feasibility

All four trainers reported that the intervention connected well with the target group, and the variation between talking and practice in the sessions was appropriate. According to the trainers, the participants handled the theory (CBT) parts well. The trainers reported that involvement of a confidant (a person who joins the participant in the individual sessions for support) during the individual sessions was of great value for the transfer to the daily life of the participants, as the trainers indicated. The adjustments that were suggested after a careful evaluation of the intervention with the trainers lead to changes that were in line with the underlying theory of the intervention. The main adjustment included increasing the trainer's and counsellor's awareness of the importance of discussing substance use in both the individual sessions and group sessions, and skipping an individual session during week five to avoid repetition. In week five participants, there is now an optional individual session or contact by phone to repeat this week with their personal changing plan.

### 3.2 User‐friendliness

Table 2 shows the results of the evaluation of the questionnaires filled out by the participants of Take it personal! The participants indicated they liked the intervention, they were satisfied with the design of the workbook, they were satisfied with their confidant, and they were content with the variation between talking and doing assignments in the intervention. They also indicated that they have learned something from the intervention.

**Table 2:** User‐friendliness (*N* = 6)

	M	*SD*	Minimum	Maximum
Liking the intervention	3.2	0.4	3	4
Learned something from the intervention	3.2	0.8	2	4
Satisfied about the counsellor	3.2	0.8	2	4
Sufficient variation in the intervention	3.3	0.5	3	4
Satisfied about the design	2.9	0.4	2	4

Note

Answer categories ranged from (1) “not right at all” to (4) “totally right.”

### 3.3 Potential effectiveness

Post‐intervention evaluation of frequency of alcohol and drug use, binge drinking and problematic alcohol and drug use indicated that level of use was lower than at pre‐intervention (see Table 3).

**Table 3:** Difference between pre‐test and post‐test in absolute numbers for alcohol, cannabis and other illicit drug use (*N* = 6)

	Pre‐test (*N* = 6)	Post‐test (*N* = 6)
Alcohol		
Last month use	6	1
Weekly use	4	1
Binge drinking	3	1
AUDIT	6	4
Cannabis		
Last month use	5	2
Weekly use	3	1
Other illicit drug		
Last month use	4	2
Weekly use	4	1
DUDIT	5	4

Abbreviations: AUDIT, Alcohol Use Disorders Identification Test; DUDIT, Drugs Use Disorders Identification Test.

## 4 DISCUSSION

The IM protocol was utilized to develop Take it personal! intervention based on theory and the results from other research that were expected to improve the likelihood of effectiveness of the intervention. The use of the IM protocol increased our understanding of the mechanisms underlying the effectiveness of Take it personal!. Evaluating the programme with a small modelling study was part of the process of the IM protocol (Bartholomew et al., 2001). This helps to investigate whether these theoretical principles have the same effect within the MID‐BIF target group as in adolescents without MID‐BIF, where effectiveness has been demonstrated in several studies (Conrod et al., 2013; Lammers et al., 2017; Mahu, Doucet, O'Leary‐Barrett, & Conrod, 2015). In addition, the modelling study supported the feasibility and user‐friendliness of Take it personal!. The trainers in this study concluded that the intervention can be conducted without difficulties. The participants felt positive about the intervention and had the feeling they have learned something from the intervention. They enjoyed the intervention, and they were motivated to complete it. This finding is promising because motivation is an important predictor of intervention completion and effectiveness (Deci & Ryan, 2000). Dropout from interventions, particularly those targeting adolescents, is a common problem, with dropout rates being as high as 50% (Connor et al., 2006). High dropout is often attributed to the adolescents disliking the intervention (Kahn, Ducharme, Travers, & Gonzalez‐Heydrich, 2009).

With regard to potential effectiveness, this modelling study revealed that a number of participants were able to reduce their use of alcohol, cannabis and illicit drugs after the intervention. Given methodological constraints, we were not able to establish whether this change was associated with the intervention; therefore, a larger scale study is needed to draw conclusions about the effectiveness of Take it personal!.

Several limitations need to be acknowledged regarding the use of IM and this study. First, IM provides a rather static picture, while developing an intervention is a dynamic and constantly changing process. Nevertheless, the intervention was developed based on sound systematic theory and evidence. Second, only a small number of participants were included in this study, making it difficult to draw conclusions. Besides, because of this small group of participants it is only generalizable to a limited extent. Third, questionnaires to assess the feasibility and user‐friendliness of the programme were bespoke questionnaires developed for this study, and therefore, there is no information about the reliability, validity and other psychometric aspects of these questionnaires. Last, information about substance use as well as the user‐friendliness of Take it personal! was obtained by self‐report questionnaires. Future research could include additional parent‐ or staff‐reported assessments in order to avoid potential self‐report bias. Concluding, Take it personal! can be a promising indicated preventive intervention designed to reduce substance use in individuals with MID‐BIF. Although, a larger scale study is needed, to draw firm conclusions about the effectiveness of Take it personal!

## CONFLICTS OF INTEREST

The authors declare that they have no competing interests.
